# Progress of Veterinary Science

**Published:** 1883-04

**Authors:** 


### Progress of Veterinary Science.

ASCARIS MYSTAX IN CATS.—There is here (Milton, Ross-shire) an epidemic among cats. Symptoms: Slight cough, abstinence from every kind of food, vomiting up worms, and death in from forty to sixty hours after first symptoms. The writer got rid of the worms in two cases with castor oil, but both cats died like others that had no medicine. In the winter of 1879 many cats died here in the same way. [Give the cats two grains of santonine in a little milk as soon as any signs of illness appear. —ED.]—*Field.*

INOCULATION OF MONKEYS WITH SYPHILIS.—Dr. Martineau has succeeded in inoculating a monkey with the serous fluid from a syphilitic chancre. The inoculation was made upon the monkey's penis, and twenty-eight days afterward two indurated chancres were found at the point of inoculation, and a few days later there was discovered an indolent enlargement of the inguinal glands.—*Weekly Medical Review.*

SHEEP FLUKE.—Reports are coming in that sheep are dying in all parts of England from attacks of that dreadful parasite, the fluke. Mr. Robert I. Wells, a good authority, states the following: "Out of 100 cases under my special treatment, in which salt, iron, sulphur, podophyllin, etc., were carefully and regularly prescribed in alternate doses, every one died, while out of the same number treated with pure vegetable charcoal in their food daily for six weeks, ninety-seven recovered, and these were very bad to commence with."—*Drovers Journal.*

ETIOLOGY OF GLANDERS.—In the Berlin Imperial Health Office, investigations have been made into the etiology of glanders, which have resulted in the discovery of the micro-organism of this disease. The newly discovered organism was subjected to "pure" cultivation for a series of generations, after which the disease was produced in a horse by inoculation with the germ thus cultivated. The investigations were carried out under the directions of Dr. Loeffler and Professor Schütz, of the Berlin Veterinary School. The discovery is of immense importance.—*Veterinary Journal.*

SWINE PLAGUE.—A letter was read at a recent meeting of the Académie des Sciences from M. Pasteur, giving an account of his progress in some researches in which he is at present engaged in the district of Vancluse. He has gone there to investigate a disease of pigs, which in one valley of the Rhone, has recently been fatal to 20,000. The disease is called "le rouge des porcs;" and M. Pasteur announces that he has discovered its cause to be a very minute organism, which, in point of size, resembles that of chicken cholera. It differs. however, in its physiological properties since it has no action on fowls, but it is fatal to rabbits and pigs, especially to white pigs. M. Pasteur has convinced himself, by experiments, that one attack affords protection against against another, and he has succeeded in inoculating pigs with organisms which have been weakened by culture, and in thus rendering these animals insusceptible.—*Veterinary Journal.*

INFLUENCE OF DIGITALINE ON THE HEART OF THE TERRAPIN.—Messrs. H. H. Donaldson and M. Warfield, of the Johns Hopkins University, made some preliminary investigations as to the action of this drug on the heart of the mammal. The results of previous observers were so contradictory and open to so many objections that a fresh study of the action of the drug upon the hearts of cold-blooded animals seemed desirable before proceeding to work with the hearts of dogs or rabbits.

The chief results are: 1. When the heart is doing normal work digitaline decreases that work. 2. There is a rough relationship between the size of the dose and the extent of the disease. 3. With small doses of digitaline the pulse rate is at first increased. 4. The diminution in the heart's work is much more dependent on the strength of the dose at any given time than on the total amount of the drug administered. A large amount given in several hours has much less effect than a smaller amount given in a few minutes. Therefore the theory that digitaline has a cumulative action takes experimental confirmation.—*Johns Hopkins University Circular.*

FIBRO-MYOMATA OF THE UTURUS OF THE SEA-LION.—At a meeting of the New York Pathological Society Dr. Liautard presented the uturus and its appendages removed from a sea-lion belonging to Barnum's collection of animals. The animal had exhibited no special symptoms of disease, and had a good appetite up to twenty.four or thirty-six hours before death. At the autopsy there were found double pneumonia and paresites in the small intestine, and at the junction of the left horn with the body of the uterus there was found a large tumor which presented the gross appearances of fibromyoma as it occurs in the human subject.

Dr. Liautard also presented a cyst containing hair which he had removed from the side of a saddle-horse, a little posterior to the left shoulder. At that point there was a movable elongated tumor, about the size of an English walnut. After making an incision through the skin the tumor was easily enucleated, and when opened it was found to contain a large amount of curly hair, mixed with sebaceous material. Another tumor, similar in feel, and about three inches in length, was removed from another part, and on opening it, it was found to contain material which presented the appearance of pus. He had been unable to find the record of any case of cyst containing hair occurring in the horse. *Medical Record.*

TEST FOR IODOFORM.—The *Medical Record*, quoting from the *Berliner Klin, Woch.*, says that in a prolonged discussion on the utility and disadvantages of iodoform, at a recent meeting of the Berliner Medicinische Gessellschaft, Dr. Steinauer had occasion to make some practical suggestions. Iodoform, he stated, when applied locally was always absorbed, though very slowly, and its action was a cumulative one. Hence it was advisable to remove the dressing after five or six days, and substitute some other antiseptic agent, as carbolic acid, thymol, or salicylic or boracic acid. Iodoform appears in the urine in the form of iodine, in combination with an alkali. Its presence can readily be determined by adding nitric acid and starch to the urine, and shaking the mixture, when the characteristic blue color appears. We have thus a simple means of estimating the amount of iodoform absorbed, and can judge whether danger of poisoning exist or not. The lethal dose in man is difficult to determine. In animals it is stated to be as follows: Guinea pigs, thirty grains; rabbits, forty-five grains, and dogs, one drachm.—*Chicago Medical Review.*

TINCTURA IODOFORMI COMPOSITA.—Under this title, Dr. G. Beck described in his "*Therapeutical Almanac*, 1880–81," a very useful formula for a solution of iodoform and iodide of potassium, which can not only be taken internally (three times a day, fifteen drops in sugared water), but is in place in all cases where the iodine treatment seems to be adapted for external application, and is capable of causing a radical disappearance of tumors of various kinds, as also of inflamed glands, etc. The original formula—iodoform, 1 part; potassii iodid., 70 parts; glycerin, 70 parts; spir. vini. rect., 100 parts—is pharmaceutically incorrect, because the iodoform is not completely dissolved in this solution. The following modification is recommended, which, while not lessening any of its effects, represents a complete solution, to which Balsam of Peru is added as a corrigens to the iodoform:

R. Iodoform.................................... 8 grms.
Balsam Peru.................................... 3 "
Solv. in spir. vin. rect........................20 "
Solutioni admisc.
Kalii iodid.....................................70 "
Ft. solut. in aquæ dest.,
Glycerin pur...............................ââ 35 "
Filter.

MALE FERN.—Dr. Frederick Manson, having observed the effects of male fern upon a dog, reports the result in the *British Medical Journal.* The dog was suffering markedly from tape worm. A drachm of the fluid extract was administered. Shortly afterward the dog began to stagger in attempts to walk, and a little later, when stood upon its feet, fell over upon its back and was unable to rise. Muscular power was completely gone. The dog remained limp and paralyzed for three days, and there was no intestinal movement during that time, in spite of cathartics. From this time onward recovery was gradual.—*Chicago Medical Review.*

OSSEOUS DEFORMITIES IN THE LOWER ANIMALS.—At the last meeting of the Pathological Society, Mr. Sutton exhibited a highly interesting series of deformed, diseased, and distorted bones which he had obtained from animals dying in the gardens of the Zoological Society. The animals which furnished the specimens were a lizard, a cursorial bird, and four monkeys; and according to the theory advanced by the exhibitor, the specimens illustrated diseased conditions, common also to man, that is to say osteomalacia, osteoporosis, cranio-tabes, and osteitis deformans. By referring all these bone changes to various phases of chronic inflammation, and by including rickets also in this classification, Mr. Sutton appeared to challenge criticism, and there were, no doubt, many members of the Society present who could have cast much light on the subject. Unfortunately, however, the reading of the paper on bone diseases, and the exhibition of specimens, was preceded by a good deal of other business, so that the President was obliged to deprecate prolonged discussion; consequently no attempt was made to appraise the true value of the facts and theories put forward by Mr. Sutton and Mr. Barwell. The subject is well worthy of the best thought and time of the Society, which would confer a great benefit, not only on pathology, but on practical medicine, if it could reduce the classification and nomenclature of bone diseases leading to deformity, to some sort of order.

DIPTHERIA OF THE BLADDER WITHOUT INFECTION IN A RABBIT.—In order to determine the time within which fibrinous casts appear in the urine in retention of this fluid, Dr. Aufrecht instituted some experiments in a rabbit. The prepuce was closed with adhesive plaster, after twenty-four hours and the urine analyzed. The process was repeated four times, each time retention being continued for twenty-four hours. Shortly after the rabbit died. Upon the mucous membrane of the bladder were found several patches of a dirty gray color, resembling exactly diphtheric false membrane. Under the microscope was seen a large number of micro-organisms, some round and some rod-shaped. The rods were either single or in pairs, and often were united in a long chain. The urethra presented a normal appearance. From this experiment Aufrecht concludes that bacteria of disease may be developed in the bladder without, as has been hitherto supposed, gaining entrance through the urethra. The condition of the canal in this case, he states, precluded any such source of infection. He thinks that this lends weight to the theory of Billroth and others, that the bacteria existing normally in the same tissues may, under proper conditions, develop into noxious organisms. But, especially, he thinks that it confirms his previously expressed opinion, that it is not the mere presence of bacteria, but their retention and development in the organs, that give rise to infectious diseases.—*Allgem. Med. Central-Zeitung.*

EQUINE SCARLATINAL VIRUS AS A PROPHYLATIC AGAINST HUMAN SCARLATINA.—The *Medical Record* of March 24th, contains an article with the above title, written by J. W. Stickler, of Orange, N. J., which places before the medical profession interesting and important views on the subject of inoculation to prevent scarlatina.

What Dr. Jenner has accomplished in modifying or preventing small pox by a subcutaneous use of vaccine, Dr. Stickler may do in scarlatina by grafting on to the human tissues the equine scarlatinal virus.

Having fully established the identity of equine with human scarlatina, he made several satisfactory experiments, first on rabbits and afterwards on twelve human patients who never had scarlatina. The result of these experiments proved the entire safety of using subcutaneous virus direct from the horse and also that this vaccination protects against the malignant form of scarlatina as developed spontaneously in man.

INTESTINAL OBSTUCTION.—In a recent number of the *Kennel Gazette* were quoted instances of dogs being killed by intestinal obstruction caused by swallowing corks. Referring to these cases a correspondent of the above journal furnishes in this month's number some valuable notes regarding the successful treatment of similar mishaps in his own kennel. The subjects in all instances were hounds fed principally on the refuse from the table of a large establishment, where the servants throw pretty well anything into the tub provided for the dogs' food; amongst other things peach stones, plum stones, and corks were constantly to be discovered. The features observable were, that the dogs became dull and heavy, disinclined to feed, and walked about with backs arched and their tails tightly held between their legs; but the most diagnostic symptom was that they turned their head to the side where the pain evidently existed, and licked it. Constipation was present, although at first shreds of mucous was passed after much straining. The treatment consisted of first passing a tube up the rectum, about six inches, and injecting, by means of an ordinary enema syringe, a pint of warm olive oil. After removing the tube the rectum was plugged with a piece of sponge the size of a goose's egg, to which was attached a string. This was retained for several hours, until the dog became very uneasy, when the sponge was withdrawn by means of the string. An evacuation followed, in which was the cork. "I have," says the writer, "had four cases, in one of which a peach stone was passed. All were treated in the same manner, and are still alive. One thing to be observed is, to pass the tube as far as possible; this must be done gradually and gently. It is quite useless to give the dog purgatives, as this only does more harm than good by causing the obstacle to be forced lower, and to become more impacted in the folds of the intestine, the object being to inflate the intestine round the obstacle, and thus to dislodge it. The same treatment I have found very useful in ordinary constipation, and always effectual without causing the distress to the dog and loss of strength produced by purgatives."—*Land and Water.*

PASTEUR AND KOCH.—Dr. Koch has published his reply to Pasteur, as he announced that he would do at the session of the Geneva Congress. Koch takes the ground that "it is not yet proved that all infectious diseases are parasitic in character, but that the parasitic character must be proved, in each case separately." As a model of how such proving should be done, he modestly cites his own experiments with the tubercle bacillus. He criticises the methods adopted by Pasteur in studying rabies and Glanders, and denies that that experimenter's conclusions regarding them have been established. Koch also claims priority in regard to the discovery of the cause of anthrax, and denies Pasteur's statements regarding the mode of propagation of that disease (*i, e.*, by the agency of earth worms.) Regarding preventive inoculations, Koch states that while some bacterial diseases may be so prevented, there are others, like gonorrhœa and erysipelas (?) against which one cannot be protected. Koch considers that Pasteur's claims for the value of preventive inoculations are exaggerated, and that his methods are cumbersome and defective. It remains now for Pasteur to say a word.—*Med. Record.*

The Newly Discovered Prophylactic Vaccine for "Rouget;" or Pneumeo-enteritis of the Pig.—M. Pasteur has just communicated to the French Academy, through M. Bouley, the results of his recent experiments upon the pig, made with a view to study the prophylaxis of the disease, which we have named in the caption to these lines, and which so deeply affects the interests of the porcine species. The practical value of this discovery is only comparable to the great benefits of charbon preventive; and like experimental deductions which gave birth to the prophylactic inoculations in the latter disease, is the offspring of the renowned culture and alternation process of the illustrious discoverer.

Rouget, or pneumeo-enteritis of the pig, is a contagious and extremely active malady, which has proven a real plague in all those countries in which pork-raising is one of the established agricultural pursuits. In the United States, the mortality from this disease amounted to 900,000 swine; in France, 20,000 of these valuable animals were killed by this pest, the loss sustained by the pork raisers, in the Western departments, Alme, amounting to three millions of francs.

M. Pasteur, and his colleagues have carefully studied the microbe which causes this disease, and have discovered that it is not the bacillus described some years ago by Dr. Klein.

They cultivated this fungus, and submitted it to the attenuation process, through which they have obtained a real vaccine, that when inoculated invests a permanent and preservative immunity upon the experimented animals. The experiments already made are numerous, and conducive enough to dispel any doubts as to the efficacy of this prophylactic vaccination. At present, preparations are being made to carry out a final and decisive test, on a large scale, next year, when the hog-pest will begin to make its ravages in the infected localities.—*Journal de Medicine et de Chirurgie Praitiques.*

Experiments on Animals with Male-fern and Kamala.—***A.***—July 28th.—Gave a pig, weighing 1½ cwt., 1 oz. Ext. Fil. Maris Liq. at 4 p. m.

July 29th, morning.—Pig has refused food and water; has passed one or two natural stools.

4 p. m.—Gave 1½ oz. Ext. Filicis, with 3 drs. Kamala. Pig died within two minutes of administration.

*Autopsy.* 30th, 8 a. m.—External surface of stomach intenseiy congested; mucous membrane, cardiac, and slightly so liver, lungs kidneys and all solid viscera intensely conjested. Bladder full of red color urine. Right heart full of blood containing a large, firm, partially adherent clot; left, empty, but containing three small clots. Death was evidently due to the first dose, the immediate cause being disturbance of clot in right heart during the animal's struggles when second dose was administered. A small quantity of second dose found its way into the trachea, probably during death struggle, the milk being found in stomach.

***B.***—July 30th.—Gave a small pig ½ oz. Ext. Fil. Maris. Death in two hours. Right heart disturbed; left, empty and contracted. Slight congestion of lungs. None of other organs.

***C.***—July 30th.—Gave a second small pig ½ oz. Ext. Fil. Liq. Refused food, became giddy, and recovered after twenty-four hours.

***D.***—Gave a dog 6 drs· fern oil, and 6 drs. more after four hours. Dog vomited once. No ill symptoms.

***E.***—Gave a dog 6 drs. with 1½ drs. Kamala. Vomiting and purging. Death in twelve hours. Right heart dilated and full of blood; left, empty. Mucous membrane, stomach and small intestines conjested in patches ; lungs congested. Other organs normal.

***F*** and ***G.***—Gave two smaller dogs ½ oz. Ext. Fil. Liq. Each dog got out during the night. but have not been seen since. The two dogs, ***D*** and ***E,*** were large Pariahs, the size of a retriever, the other two about half that size. —*The Veterinarian.*

THE MOVEMENT OF BOWELS IN HEALTH AND DISEASE have lately been carefully studied by Nothnagle in the *Deutsche Zeitschrift für Kin Med,* the conclusions he has arrived at are of great interest. The abdomen of animals was opened under an anæsthetic, and the body placed in a one-half per cent. saline solution kept at a temperature of 100.2 Fahr. The result of this was that all movements in the bowels, with the exception of the duodenim ceased, but if the intestines were dilated with fluid or gas violent peristaltic action resulted. The contractions are all from before backwards, anti-peristalsis never occurs in a normal intestine. The effects of rectal injection showed that warm water remained where it was thrown, never being carried forwards, but it increased slightly the peristaltic actions. Iced cold water caused contraction of the gut and passed a short distance forward ; olive oil acted in a similar manner, but a strong solution of chloride sodium was carried up by forward contraction as high as the cæcum, taking the fœces with it. Solutions of nitrate potash, bromide potasium and weak ones of sulphate of copper behaved in a similar manner.

Ligaturing a small intestine produced, first of all, contractions of the bowel anterior to the ligature, but these shortly ceased, and the part became paralyzed. By artificially inducing enteritis it was found that the peristaltic movements were greatly increased, and the inflamed portion became filled with fluid, probably inflammatory products, but after the lapse of forty-eight hours the condition was different, the bowel was now motionless.

In intussesception, Nothnagle noted that it occurred from before backwards, a contracted portion passing into a dilated one. An invagination of the colon was distinctly removed by the antiperistaltic movement brought about by the injection per rectum of chloride of sodium. The influence of morphia on the movements is most interesting, a small quantity prevents a salt of soda causing its usual contractions, but a large quantity of morphia be injected. Then the salt of soda not only produces its usual effect, but in a much more violent manner.

The results of these experiments are of great practical importance, rectal injections of chloride of sodium may be of great service in impaction of the bowels and intussusception. The paralyzed ligatured bowel shows the injurious effects of purgatives in intestinal obstruction, these, by increasing the peristalsis, carry the contents of the bowel towards the seat of obstruction, and more quickly bring about the paralysis which follows distension. The movements in interitis are more instructive. and it would appear that in the early stages of this disease the contents of the bowel are moved with increased rapidity through the inflamed portion. Morphia seems to act on the bowels as digitalis does on the heart, namely, stimulates or paralyses the inhibitory nerve.—*Quarterly Journal of Veterinary Science in India.*

MEDICINE AS PRACTISED BY ANIMALS.—M. G. Delaunay, in a recent communication to the Biological Society, observed that medicine, as practised by animals, is thoroughly empirical, but that the same may be said of that practiced by inferior human races, or, in other words, by the majority of the human species.

Animals instinctively choose such food as is best suited to them. M. Delaunay maintains that the human race also shows this instinct, and blames medical men for not paying sufficient respect to the likes and dislikes of the patients, which he believes to be a guide that may be depended on. Women are more often hungry than men, and they do not like the same kinds of food; nevertheless, in asylums for aged poor, men and women are put on precisely the same regimen. Infants scarcely weaned are given a diet suitable to adults, meat and wine which they dislike and which disagree with them. M. Delaunay investigated this question in the different asylums of Paris, and ascertained that children do not like meat before they are about five years of age. People who like salt, vinegar, etc., ought to be allowed to satisfy their tastes. Lorain always taught that with regard to food, people's likings are the best guide.

A large number of animals wash themselves and bathe, as elephants, stags, birds, and ants. M. Delaunay lays down as a general rule, that there is not any species of animal which voluntarily runs the risk of inhaling emanations arising from their own excrement. Some animals defæcate far from their habitations; others bury their excrement; others carry to a distance the excrement of their young. In this respect they show more foresight than man, who retains for years excrement in stationary cesspools, thus originating epidemics.

If we turn our attention to the question of reproduction, we shall see that all mammals suckle their young, keep them clean, wean them at the proper time, and educate them; but these maternal instincts are frequently rudimentary in women of civilized nations. In fact, man may take a lesson in hygiene from the lower animals.

Animals get rid of their parasites by using dust, mud, clay, etc. Those suffering from fever restrict their diet, keep quiet, seek darkness and airy places, drink water, and sometimes even plunge into it. When a dog has lost its appetite, it eats that species of grass known as dog's grass (*chiendent*), which acts as an emetic and purgative. Cats also eat grass. Sheep and cows, when ill, seek out certain herbs. When dogs are constipated they eat fatty substances, such as oil and butter, with avidity, until they are purged. The same thing is observed in horses. An animal suffering from chronic rheumatism always keeps as far as possible in the sun. The warrior ants have regularly organized ambulances. Latreille cut the antennæ of an ant, and other ants came and covered the wounded part with a transparent fluid secreted from their mouths. If a chimpanzee be wounded, it stops the bleeding by placing its hand on the wound, or dressing it with leaves and grass. When an animal has a wounded leg or arm hanging on, it completes the amputation by means of its teeth. A dog on being stung in the muzzle by a viper, was observed to plunge its head repeatedly for several days into running water. This animal eventually recovered. A sporting dog was run over by a carriage. During three weeks in winter it remained lying in a brook, where its food was taken to it; the animal recovered. A terrier dog hurt its right eye; it remained lying under a counter, avoiding light and heat, although habitually he kept close to the fire. It adopted a general treatment, rest, and abstinence from food. The local treatment consisted in licking the upper surface of the paw, to which he applied the wounded eye, again licking the paw when it became dry.

Cats also, when hurt, treat themselves by this simple method of continuous irrigation. M. Delaunay cites the case of a cat which remained for some time lying on the bank of a river; also that of another cat which had the singular fortitude to remain for forty-eight hours under a jet of cold water.

Animals suffering from traumatic fever treat themselves by the continued application of cold, which M. Delaunay considers to be more certain than any of the other methods.

In view of these interesting facts, we are, he thinks, forced to admit that hygiene and therapeutics, as practised by animals, may, in the interests of psychology, be studied with advantage. He could go even further, and say that veterinary medicine, and perhaps human medicine, could gather from them some useful indications, precisely because they are prompted by instinct, which are efficacious in the preservation or the restoration of health.—*British Medical Journal.*

FORM OF THE PULSE WAVE.—Messrs. W. H. Howell and F. Donaldson,

Jr., have recently made some observations upon the form of the pulse wave and the mean arterial pressure in a dog with patent *Ductus Arteriosus*. The dog was about to be killed when it was noticed that its cardic action was abnormal. Prof. Donaldson examined the animal, and found the pulse rate greater and the cardic impulse more marked than in normal dogs; also the apex of the heart extended much farther to the left, showing marked hypertrophy. Over the whole cardic region he observed a loud, rasping, cardic murmur, with the maximum of intensity over the base; also a slight murmur with the second round. Subsequent experiments made on the dog showed a normal arterial pressure, and no abnormality in sphymo-graphic tracings except an unusually marked dicrotism.

Prof. Tiffany made the post-mortem on the animal, and found that the *ductus arteriosus* had remained open, its calibre being at least equal to that of either pulmonary artery.

It is a fact of much interest that with such free communication between aortic and pulmonary circulations so good an average arterial pressure (as measured in the femoral artery) was maintained. It can only be explained by assuming (1) a compensatory increase in the force of the heart-beat: (2) an increase in the resistance of the pulmonary circuit; or (3) an increase in the total bulk of blood in the body. Whichever of these agencies was the effective one, the case is one of great interest as indicating the power of the animal body to adapt itself to unusual conditions of life. So far as the authors could discover only one similar case was previously on record.—*John's Hopkin's University Circulars.*

The Prevention of Hydrophobia.—In 1862 Bourrel suggested that when the permanent teeth of a dog are well grown, the incisors and canine teeth can be blunted so as to render it impossible for a dog to inflict wounds on men or animals which might lead to inoculation with the virus of rabies. At the time his suggestion was much ridiculed, one journal suggesting that all dogs should be provided with false teeth. The question has been revived in the *Medical Press and Circular*, January 17th, 1883, by "a member of the Sanitary Institute of Great Britain."

"In general," says M. Bourrel, "it is a sharp pinching produced by the front teeth that causes inoculation; the skin is torn, or the bite draws blood. By blunting, or resection, sixteen obtuse surfaces are substituted for sixteen sharp points. Sporting dogs in the habit of tearing the game have been prevented from doing so by this measure, while the furious disposition of some dogs, such as watch dogs, which render them dangerous to every one, was softened, and brutes which would have to be destroyed were consequently allowed to live. Terriers have not ceased to kill rats after this blunting; they have only lost their power to kill cats, which is a happy result. The same operation disarms those bull-dogs that certain individuals have the discreditable passion of exciting to fight; pet dogs have been operated upon without any inconvenience."

Pasteur on Rabies.—At a recent meeting of the Académie de Medécine M. Boulay communicated, in the name of M. Pasteur, a series of conclusions regarding rabies at which the distinguished investigator has arrived. The first two enunciated the familiar truths that the dumb madness and the furious madness, and, in short, all varieties of rabies, are caused by the same virus, and that the symptoms of rabies are extremely variable. It is assumed that the characters of the several cases depend on the points in the nervous system at which the effect of the virus is chiefly localized. In the saliva of rabid animals the virus is associated with several kinds of organisms, and the inoculation of the saliva may cause death in these ways, by means of the special salivary organisms, by excessive suppuration, and by rabies. The medulla oblongata of the human subject, as well as that of animals after death by rabies, is always virulent, and the virus is also found in all parts of the brain, and it persists even after putrefaction has set in. There are two methods of inoculation by which tbe period of incubation of rabies may be greatly shortened and the disease produced, not only rapidly but with certainty; one is by the injection of virus in the blood; the other is by trephining the skull, and placing the virus in the arachnoid cavity.

Rabies then come on at the end of six, eight, or ten days. M. Pasteur has met with some cases of the "spontaneous cure" of rabies, but only in cases in which the disease did not develope beyond the initial stage. In such a case, in which the early symptoms passed away, the disease has been known to return at the end of a certain time—as two months—and then to run the ordinary acute and fatal course. Mention is also made of the cases of three dogs inoculated in 1881; two quickly died from rabies; the third, after manifesting the early symptoms, recovered. The latter animal was inoculated by trephining in 1882 on two separate occasions, but without effect. M. Pasteur asserts that he now possesses four dogs which will not contract rabies, whatever the method of inoculation adopted or the proved virulence of the material employed. These facts he believes to be the first step toward the discovery of a method of the prevention of rabies by its inoculation. He confesses, however, that the end seems to be at present far distant.—*London Lancet.*

THE GERM OF HOG CHOLERA.—At a meeting of the Academy, held Dec. 4, 1882, Messrs. Pasteur and Thuillier presented the results of their researches on mal-rouge of Swine.

1st. Mal-rouge of Swine is produced by a special microb easily cultivated outside of the bodies of animals. It is very tenuous and resembles most that of the chicken cholera. Its form is also that of the figure 8. It has no effect on chickens but kills rabbits and sheep.

2. Inoculated in a pure state in the pig in almost inappreciable doses, it quickly produces disease and death with the characteristics habitual in spontaneous cases. It is especially fatal to the white race called perfected.

3. Dr. Klein published in London, in the year 1878, an exhaustive work on the Rouget which he calls pneumo-enteritis of the pig; but this author is mistaken as to the nature and properties of the parasite. He has described as a microb of the mal-rouge a Bacille (Samphire ?) with spores more voluminous even than the bacteria of anthrax, very different from the true microb of Rouge. The Samphire of Dr. Klein has besides no relation with the etology of this disease.

4th. After having proved that the disease does not re-appear, the authors have practiced successfully inoculations preventing it from being fatal.

5th. Though new experiments are yet necessary, it is to be hoped that in a short time vaccination by the attenuated microb of the Rouget will become the safeguard of herds of Swine.

